# Long-term sustainability of biogas bubbles in sand

**DOI:** 10.1038/s41598-020-69324-0

**Published:** 2020-07-29

**Authors:** Xiaoying Hu, Dandan Li, Erxing Peng, Zheng Hou, Yu Sheng, Yaling Chou

**Affiliations:** 1 Lanzhou University of Technology, Lanzhou, 730050 Gansu China; 2 State Key Laboratory of Frozen Soil Engineering, Northwest Institute of Eco-Environment and Resources, Chinese Academy of Sciences, Lanzhou, 730000 Gansu China

**Keywords:** Civil engineering, Mechanical properties

## Abstract

Desaturation is a new method to mitigate liquefaction of sand. It aims to prevent liquefaction by generating gas/air in the pores of fully saturated sands, and biogas is one of the most suitable gas. In order to evaluate the long-term sustainability of biogas bubbles, a series sustainability test on biogas bubbles in pores of sand was conducted with a one-dimensional device under hydrostatic condition, hydraulic gradient flow condition and horizontal excitation condition. The variation trend of the retention of biogas bubbles in the pores of soil under the aforementioned conditions was analyzed. Test results indicated that after 72 weeks of monitoring sand samples, biogas bubbles existed stably in the pores of soil under hydrostatic conditions. In hydraulic gradient flow, the stability under upward seepage flow showed a similar trend to that of downward seepage flow. When the hydraulic gradient was constant, the degree of saturation increased in a certain period and finally remained constant. When the hydraulic gradient increased by 0.1, 0.2, 0.3, 0.4, and 0.5, the degrees of saturation increase were 0.8%, 11.5%, 0.5%, 0.1%, and 0%, respectively. After 41,200 cycles with different accelerations, the degree of saturation of the sample increased slightly, and the biogas bubbles basically remained stable.

## Introduction

As the plates beneath the Earth’s crust squeeze and collide with one another, seismic fault zones become active^1–4^, and seismic activities are distributed in a wide range, thereby causing serious geological disasters. Every strong earthquake results in considerable damage^5–9^. Liquefaction, in particular, causes great harm to people's properties and lives^10,11^. Liquefaction refers to the mechanical process of saturated fine sand losing its strength instantaneously and changing from solid to liquid under the action of seismic force. Liquefaction mostly occurs in loose saturated sand^12,13^. Therefore, dealing with liquefied subgrade simply, economically, and effectively has become a key technical problem in infrastructure construction.

Therefore, a new desaturation method was proposed to treat liquefiable sand^14–18^. In accordance with the gas injection method, the new method can be divided into air injection^19–21^, water electrolysis^22^, chemical bubble formation^23,24^, drainage–recharge, and biogas bubbles^25^. Among these methods, biogas bubbles are one of the most suitable materials for saturation reduction. Biogas bubbles method is a kind of desaturation method which uses denitrification of denitrifying bacteria to reduce nitrate ions in pore water to nitrogen. Denitrification is a process in which denitrifying bacteria gradually reduce nitrate to nitrogen under anoxic conditions, and the final product of denitrification is nitrogen. Biogas bubble method has the following advantages: First, bubbles are generated in the soil in the original position and are evenly distributed in the soil. Second, the microbial reaction is slow, and the process is easily controllable. Third, the nitrogen produced by microbial denitrification^26^ has the characteristics of stable chemical properties and low solubility.

In a field test, Okamora et al.^19,27^ investigated the ground of a site improved 26 years ago by a sand compaction pile and concluded that bubbles survived for 26 years. At the same time, they indicated that if the degree of saturation is less than 90% at the end of the construction of a sand compaction pile, the unsaturated phenomenon in the soil will remain for a long time. In laboratory tests, Yegian and Eseller proposed a method of using electrolytic water to produce hydrogen and oxygen^28^ in saturated soil to reduce its saturation and thereby improve the anti-liquefaction strength of soil^22^. He et al.^29^ studied the stability of biogas bubbles under seepage conditions by using a one-dimensional model sand column and found that some bubbles would flow out together with pore water under seepage conditions and thus reduce the degree of saturation effect. The stability of biogas bubbles under hydrostatic and horizontal excitation has not been researched; only the stability of one-dimensional seepage under a fixed hydraulic gradient has been studied.

Previous studies primarily focused on the stability of air bubbles or hydrogen and oxygen bubbles produced by electrolysis. They also studied the stability of biogas bubbles under one-dimensional short-term seepage with a single hydraulic gradient. However, the long-term sustainability of biogas bubbles in soil under hydrostatic hydraulic gradient flow and horizontal excitation is unclear and has not been systematically studied. Therefore, the long-term sustainability test of biogas bubbles in the sand under hydrostatic conditions, different hydraulic gradient flow conditions, and vibration conditions were conducted. The results can reflect the effective time of a single reinforcement using biogas bubbles, which also provide a theoretical basis for application of the construction using desaturation method.

## Materials and methods

### Materials

#### Sand

**Figure 1:**
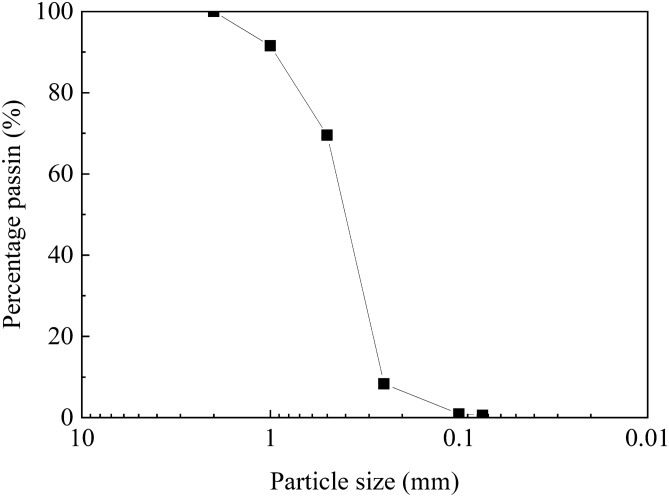
Particle-size distributions curve of sand sample.

The sand used for this study is source from Nanjing in Jiangsu Province. Conventional tests of specific gravity and particle classification of sand obtained that the specific gravity of sand was 2.68, and the maximum and minimum void ratios of sand were 0.946 and 0.428 after the conversion of corresponding minimum and maximum dry densities directly measured. Figure 1 shows the particle size distribution curve of the sand, and the gradation parameters of the sand are summarized in Table 1.

**Table 1:** Gradation constants of sand.

*d*_10_ (mm)	*d*_30_ (mm)	*d*_50_ (mm)	*d*_60_ (mm)	*C*_u_	*C*_c_
0.27	0.33	0.42	0.46	1.71	0.88

In Table 1, *d*_10_ is the effective diameter, *d*_30_ is the medium diameter, *d*_50_ is the average grain diameter, and *d*_60_ is the constrained diameter, *C*_u_ is the non-uniformity coefficient, *C*_c_ is the curvature coefficient. It can be seen from Fig. 1 and Table 1 that the content of coarse sand group is more than 50%, without gravel and fine grains, and *C*_u_ < 5 and *C*_c_ < 1. According to Reference^30^, it is named as poorly graded sand.

#### Bacteria and denitrifying cultivation

The bacteria strain used was P. *stutzeri*^31^ (German Collection of Microorganisms and Cell Cultures [DSMZ]^32^ 5190). It is necessary to note that "5190" is the serial number of the bacteria in the official website of DSMZ. For denitrifying cultivation of the bacteria, the medium was composed of the following components: 0.2 g of magnesium sulfate heptahydrate, 2 g of potassium nitrate, 1 g of dipotassium phosphate, 5 g of sodium citrate dihydrate, and the addition of distilled water a total volume of 1 L.The equation for denitrification of *P. stutzeri* DSMZ 5,190 is as follows:

$$ 5{\text{C}}_{5} {\text{H}}_{7} {\text{O}}_{5} {\text{COO}}^{ - } + 18{\text{NO}}_{3}^{ - } \to 30{\text{CO}}_{2} + 9{\text{N}}_{2} \uparrow + 6{\text{H}}_{2} {\text{O}} + 23{\text{OH}}^{ - } $$

In order to verify the stability of nitrogen in the pores of soil, digital camera was used to monitor the nitrogen.

### Methods

#### Hydrostatic condition

**Figure 2:**
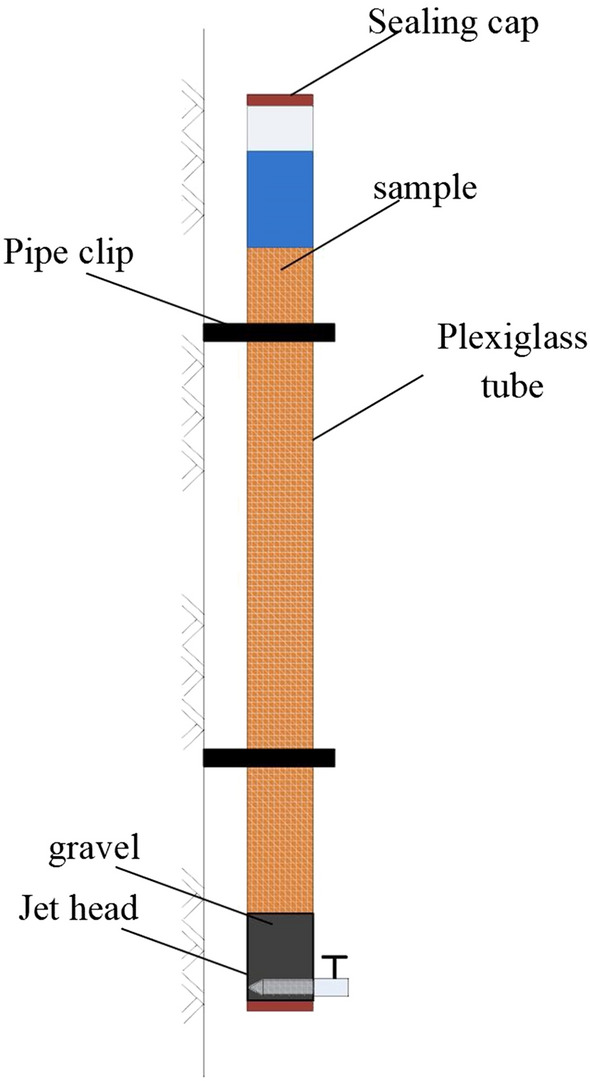
Setup for testing sustainability of air under hydrostatic conditions.

The setup for testing of the long-term sustainability of biogas bubbles under hydrostatic conditions is shown in Fig. 2. First, full saturated sand samples were prepared in a plexiglass tube. The height of the sample in the test was 0.8 m. The height of the plexiglass tube was 1.0 m, the outer diameter was 10.5 cm, and the inner diameter was 10 cm. A certain amount of bacterial suspension was added to the bottom of the model, and then sand was poured evenly from the top of the model. The sand sample was compacted once for every 10 cm rise, and the same compaction work was used for each compaction. The sand was below the liquid level all the time. The sample reached a predetermined height, and the top of the sample was tightly closed with a 1 cm-thick piece of liquefied paraffin to avoid evaporation. After denitrification, the height of the top surface of the liquefied paraffin was measured, and the initial degree of saturation of the sample was calculated.

The calculation formula is as follows:

1 $$ V_{a} = \Delta h \times A $$

2 $$ S_r = (V_{v} - V_{a} - V_{ao} )/V_{v} $$

where $S_r$ is the sample’s degree of saturation, $V_v$ is the pore volume, $V_a$ is the bacterial gas production, $V_{ao}$ is the initial gas content of the sample, $\Delta h$ is the variation of water thickness on the top surface of the sample, and $A$ is the cross-sectional area of the sample.

Second, bubble sustainability was observed. In the first three months, the data were recorded daily; weekly measurements were performed for the rest of the observation period, and the measuring temperature was 20 °C.

#### Hydraulic gradient flow condition

**Figure 3:**
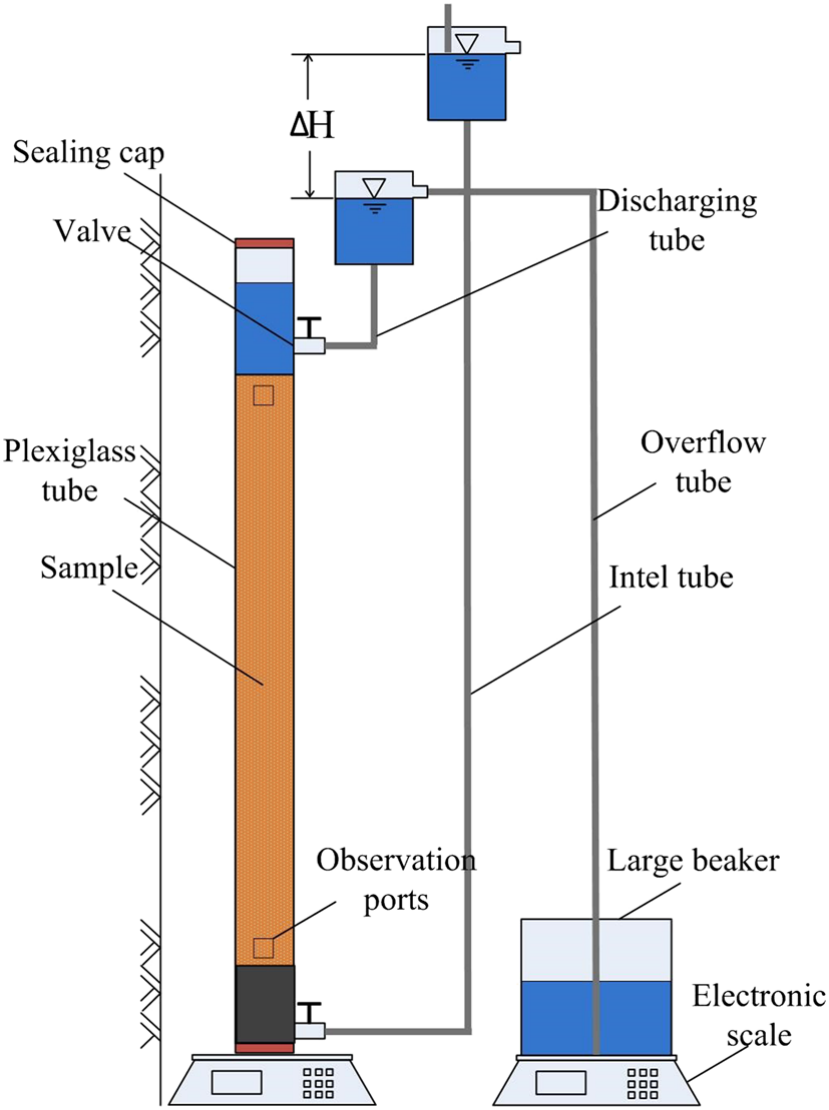
Setup for testing sustainability of air hydraulic gradient flow conditions.

A schematic of the experimental device for the evaluation of biogas bubble stability under different seepage conditions is shown in Fig. 3. On the basis of the hydrostatic condition test, a bucket, water head, and electronic scale were added. The inlet and outlet water tubes and overflow tubes were plastic tubes with diameters of 1–2 cm. The precision of the electronic scale was 0.5 g, and the range was 30 kg. After the preparation of the sample containing biogas bubbles, the plexiglass tube was filled with a bacterial suspension, and the top of the plexiglass tube was sealed with a seal cap. The stability of the biogas bubbles in the soil under different hydraulic gradients was measured by a constant head test.

Two types of bubbles were used: accumulated biogas bubbles and dissolved biogas bubbles. Sample saturation could be measured by two methods. In the first method, if biogas bubbles come out in a gas phase, they accumulate at the top of the tube. Then, the volume of this portion could be measured. The reduction in gas volume $\Delta V_a$ could be measured as well. Thus, sample saturation could be calculated. In the second method, if biogas bubbles come out dissolved from the samples, water would occupy the space vacated by the biogas bubbles. The reduction of biogas volume in this form could be evaluated by measuring the increase in the weight of the sample. The seepage discharge was measured by an electronic scale, and the coefficient of permeability of the soil sample was calculated on the basis of the hydraulic gradient. The equation is

3 $$ \Delta V_a = \Delta m/\rho $$

where $\Delta m$ is the change in sample mass and $\rho$ is the density of water.

Hydraulic gradients of 0.1, 0.2, 0.3, 0.4, and 0.5 were adopted. The test was conducted by increasing the hydraulic gradient gradually. Data were read every 12 h until the degree of saturation was no longer changed for 24 h. The void ratio is calculated by measuring the height of sand sample.

#### Horizontal excitation condition

**Figure 4:**
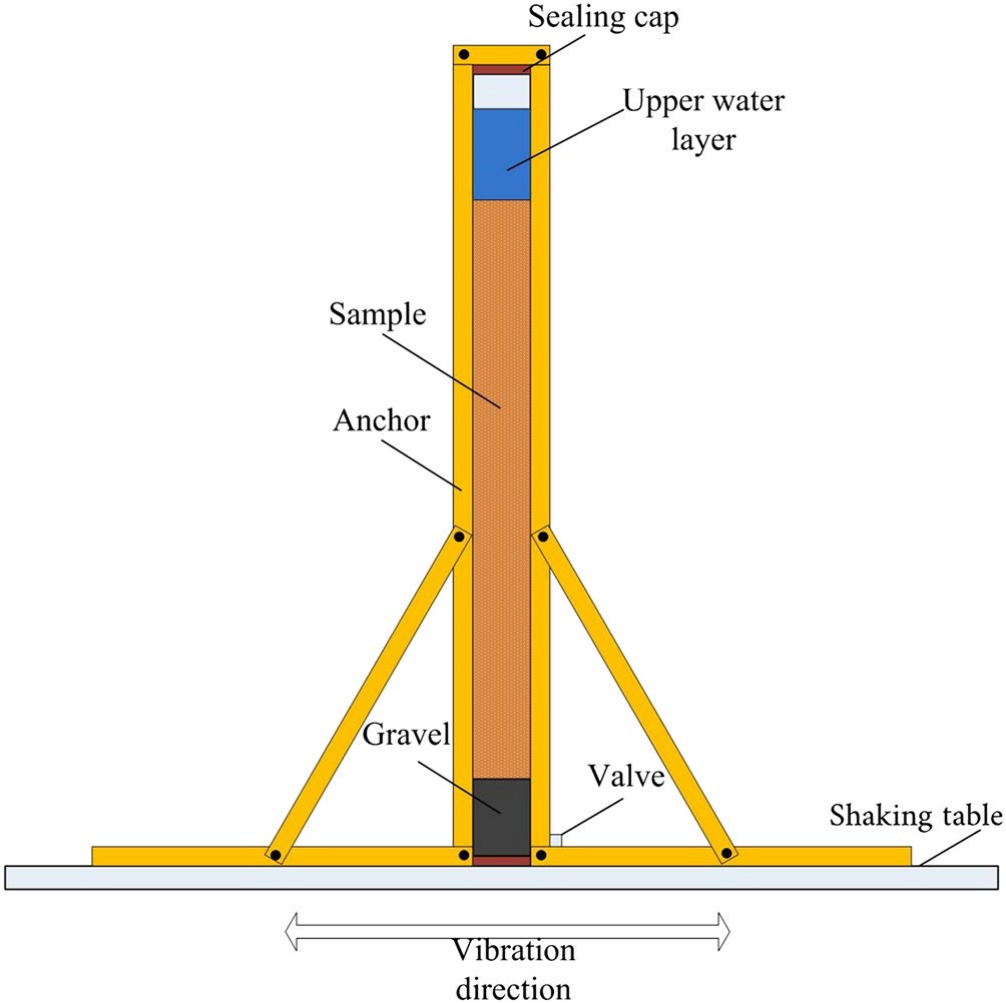
Experimental setup for testing air-entrapped specimens under horizontal excitation.

The setup is schematically illustrated in Fig. 4. The partially saturated sample preparation method is the same as the hydrostatic condition. Under seismic load, if the biogas bubbles overflow in the sample, the upper water layer will drop. Measuring the thickness change of the upper water layer and calculating the volume loss of the bubbles are necessary. Given that volume strain will occur when the sample is under seismic load, the bubble overflow amount is not the only factor that determines the soil degree of saturation. Furthermore, the height of the sample should be measured to obtain the volume strain. Therefore, on the basis of biogas overflow amount and the volume strain of the sample, the degree of saturation can be calculated. In the hydrostatic condition test, the test equipment still needed a small model shaking table, wooden frame, and other equipment. The vertical plexiglass tube was placed on a small shaking table. A wooden block was used to enable the tube and table to vibrate simultaneously and thereby eliminate any possible redundant rocking motion. The cyclic loading was sine waves with 2 Hz. The amplitudes of the sine waves were 0.5, 1.0, 1.5, and 2.0 m/s^2^. After loading 300 cycles 10 times for each level of load, 10,000 cycles were loaded 10 times. The specimen was loaded with 41,200 cyclic loads. The calculation method for the degree of saturation was consistent with the hydrostatic condition.

## Results and analysis

### Stability of biogas under hydrostatic condition

**Figure 5:**
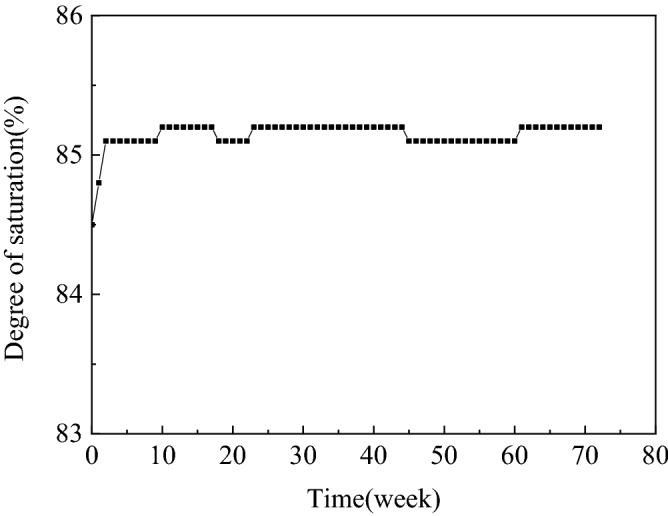
Long-term monitoring of entrapped air under hydrostatic conditions.

Figure 5 shows the monitored and measured data for 72 weeks. The initial degree of saturation of the sample was 84.5%. After 72 weeks, the degree of saturation of the sample was 85.1% and increased by 0.6%. The period of the degree of saturation growth mainly occurred in the first two weeks possibly because a small portion of the bubbles on the upper part of the sample escaped from the soil due to external disturbance. Yegian et al.^33^ prepared unsaturated samples that contained air bubbles by drainage–recharge, after 442 days, the degree of saturation increased from 82.1% to 83.9%, which indicated a 1.8% increase. According to the above test results, nitrogen bubbles produced by microorganisms are more stable than air bubbles. A possible reason for this result is the low utilization of nitrogen by microorganisms; 0.6% is caused by pure disturbance, whereas oxygen in air bubbles produced by the drainage and water injection method can be used by microorganisms, resulting in the reduction of gas and the increase of the degree of saturation by 1.8%.

### Stability of biogas under hydraulic gradient flow

#### Downward flow

**Figure 6:**
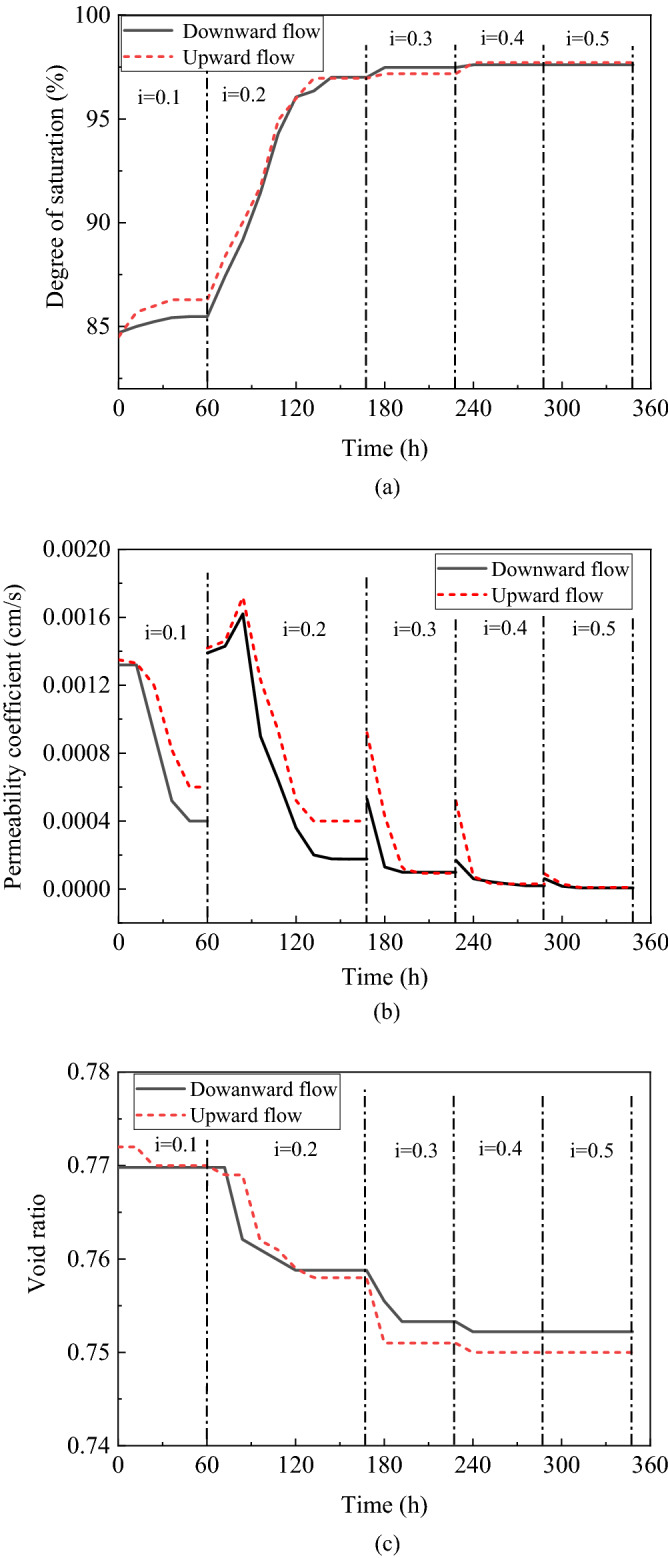
The change of main parameters.

Figure 6a–c show the degree of saturation, coefficient of permeability, and void ratio under different seepage conditions.

The experiment showed a similar trend in the change in the main parameters under the condition of upward flow and downward flow. Figure 6 illustrates the downward flow as an example. As Fig. 6a shown, all degrees of saturation increased under a hydraulic gradient of less than or equal to 0.4; when the hydraulic gradient was 0.5, the degree of saturation of 97.6% did not rise. The degrees of saturation of soil did not increase all the time, they increased for a certain period and finally remained constant when the hydraulic gradient was constant.

The instability of the samples mainly occurred when the hydraulic gradient was 0.2. This result agreed with “gas bubbles in sand are stable at hydrostatic condition, while water flow could affect the stability of gas bubbles in sand” by HE JIA (2013)^34^. In this situation, degree of saturation increased from approximately 85.5% to 97.0%, and the rate of increase was higher than that for the other head gradients. When the hydraulic gradient increased by 0.1, 0.3, 0.4 and 0.5, the degrees of saturation increased by 0.8%, 0.5%, 0.1%, and 0%, respectively.

Figure 6b shows the change in the void ratio with time under the condition of downward flow. Under a hydraulic gradient flow of 0.2–0.4, the void ratio decreased with time and finally remained constant. A small decrease in void ratio was observed, dropping from approximately 0.769 to 0.752 (the relative density increased from 38.1% to 41.8%).

Under flow conditions with a hydraulic gradient of approximately 0.2, a change in void ratio from 0.770 to 0.758 was observed in approximately 108 h. The void ratio remained constant in 24 h and then decreased from 24 to 108 h.

Figure 6c shows the change of the coefficient of permeability with time under the condition of downward flow. The coefficient of permeability decreased with the passage of time and finally remained constant when the hydraulic was constant. Two main reasons explain the decrease of the coefficient of permeability: the aggregation of bubbles and bacterial plugging. Microorganisms can plug voids and reduce permeability.

**Figure 7:**
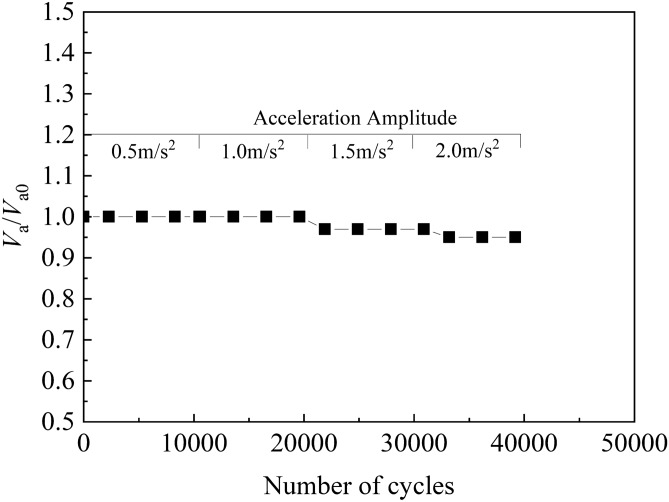
Normalized volume of air during uniform cyclic tests.

Under flow conditions with a hydraulic gradient of 0.2, a change in the coefficient of permeability from 0.00132 to 0.000176 cm/s was observed in approximately 108 h. The coefficient of permeability peaked in 24 h and then decreased from 24 to 108 h. The coefficient of permeability first increased and then decreased only when the hydraulic gradient was 0.2. The increase could be attributed to the bubbles flowing easily in the soil and some of them aggregating away from the sample. Observation points were arranged for the test at a distance of 15 cm away from the bottom of the sample, as shown in Fig. 7. The decrease could be attributed to bacteria moving in porous media under the effect of seepage from 24 to 108 h.

### Stability of biogas under excitation condition

Figure 7 shows the relationship between *V*_a_/*V*_ao_ and the number of cycles. *V*_ao_ is the initial air volume in the sample, and *V*_a_ is the volume of biogas.

As shown in the Fig. 7, sine waves were applied for 10,300 cycles. When the amplitude of acceleration was 0.5 and 1.0 m/s^2^, *V*_a_/*V*_ao_ was consistently 1.0. However, when the acceleration amplitude was 1.5 and 2.0 m/s^2^, *V*_a_/*V*_ao_ after vibration was 0.97 and 0.95, respectively. The possible reason is that a small number of bubbles in the upper part of the sample were unstable and overflowed in the soil under vibration.

**Figure 8:**
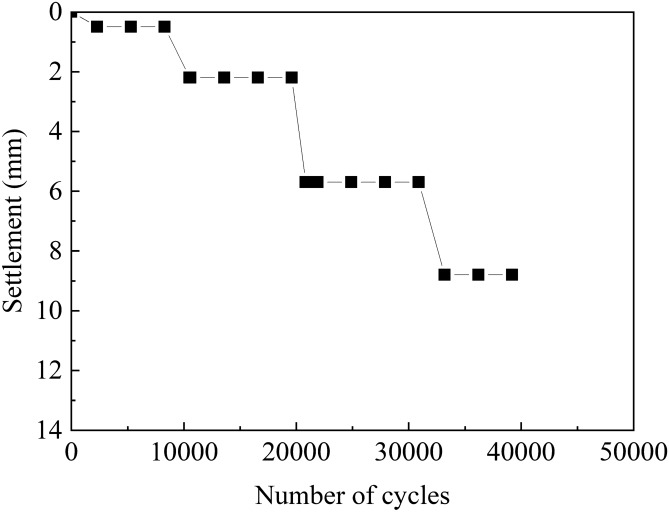
Settlement of the sand specimen during uniform cyclic tests.

Figure 8 shows the variation of sample settlement with the number of cycles. A large amplitude of acceleration caused a large total sample settlement. When the acceleration was 0.5, 1.0, 1.5, and 2.0 m/s^2^, the sample settlement was 0.5, 2.2, 5.7, and 8.8 mm, respectively. At the end of the test, the maximum volumetric strain of the sample was only 1.1%.

**Figure 9:**
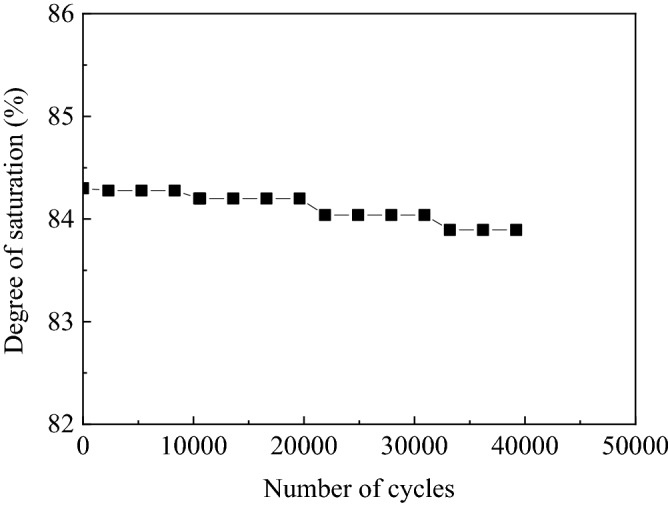
Variation of degree of saturation during uniform cyclic tests.

Figure 9 shows the variation of the sample’s degree of saturation with the number of cycles. After 41,200 cycles, the degree of saturation of the sample decreased from 84.3% to 83.8%. Although bubbles overflowed when the sample was vibrating, the overflow amount was considerably small (Fig. 7). However, during the test, due to the sample settlement, the relative compactness increased, whereas the sample degree of saturation decreased. In conclusion, after 41,200 cycles, a small amount of bubbles overflow and sample saturation drops from 84.3% to 83.8%. Eseller-Baya et al.^35^ used a small shaking table to test the bubble sustainability of cylindrical sand samples treated by drainage–recharge with an initial saturation of 85.7% under vibration conditions. Under 1 g horizontal excitation and after 300,000 cycles, the loss of air volume was less than 1% of the original air volume previously introduced in the sand specimen. The result of reference^35^ is consistent with this paper, as same as air bubbles, biogas bubbles can be stable existing in pore of sand under excitation condition.

## Discussion

The test results show that the reason for the instability of biogas bubbles is seepage condition. In the seepage condition, degrees of saturation increased, but the coefficient of permeability decreased with time when the hydraulic gradient remained the same. In the test, no biogas bubble aggregation occurred on top of the plexiglass tube, and no biogas bubbles were observed to flow away from the sample. Thus, the decrease in biogas bubbles was only due their dissolution.

**Figure 10:**
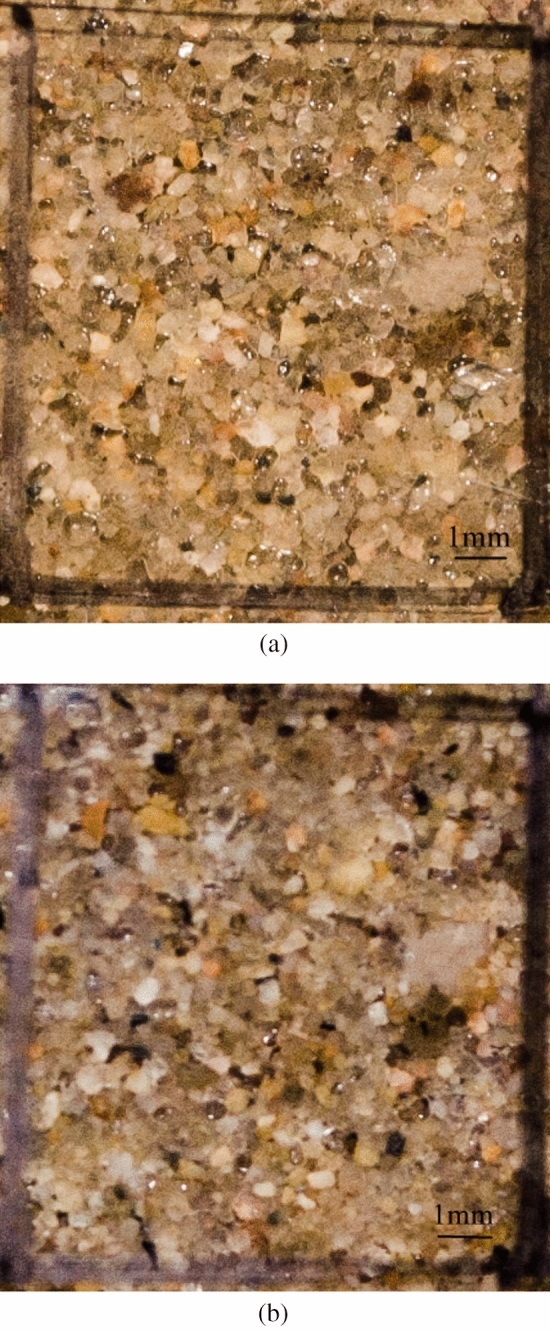
The change of bubbles.

**Figure 11:**
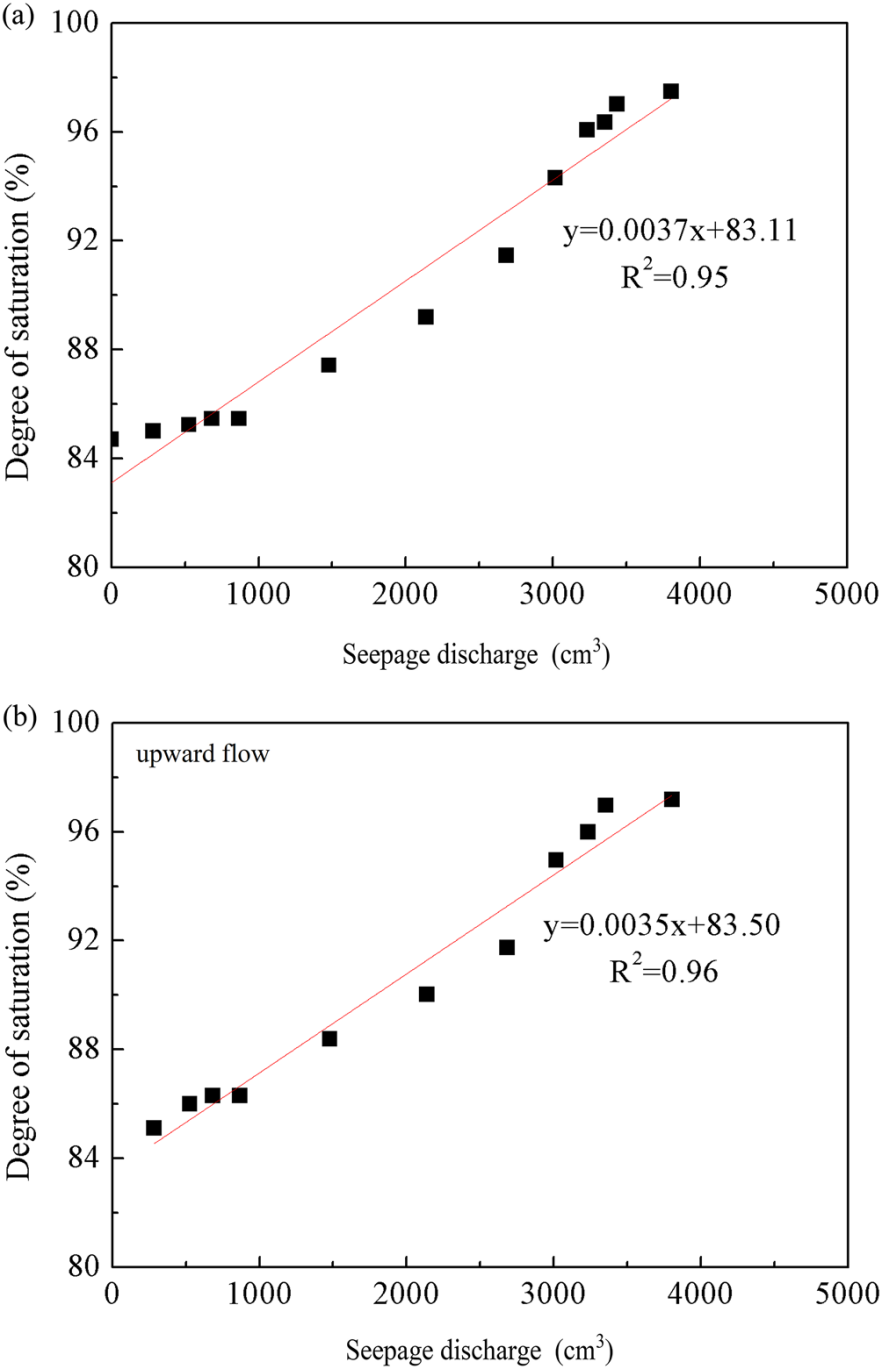
The relationship between degree of saturation and seepage flow under seepage condition.

As water flowed through the pore space, the partial biogas bubbles gradually dissolved and were lost (Fig. 10). On the basis of the test results, the relationship between the degree of saturation and the seepage flow was set up (Fig. 11). The linear relationship also verified the fact that biogas bubbles were removed in the dissolved phase.

When the hydraulic gradient was constant, seepage did not occur all the time; the degree of saturation did not increase to 100%. The major reason for the decrease in permeability was bubble plugging. Two processes related to biogas bubble stability occurred concurrently under seepage conditions: biogas bubbles removed in the dissolved phase and biogas bubbles that aggregated. The previous test results show that the increase in the degree of saturation was due to the biogas bubbles removed in dissolved form. When the biogas bubbles and bacteria gradually plugged the channel, a decrease occurred in the coefficient of permeability and seepage flow; the degree of saturation tended to be stable. The two processes inhibited each other.

In first process, the bubbles were removed with the seepage after their dissolution, and the particle soil was rearranged, resulting in the increased compactness of the sand and reduced soil permeability. In this test, the relative compactness of the soil increased slightly by 3.7% (from 38.1 to 41.8%) and 4.7% (from 37.5 to 42.2%) under downward flow and upward flow, respectively. According to the literature, when the relative density of graded soil increased from 38.0 to 43.0% and the increment was 5.0%, the coefficient of permeability decreased from 0.0014 to 0.0012 cm/s. Thus, the increase in relative compactness was not the main reason for the decrease in the coefficient of permeability.

**Figure 12:**
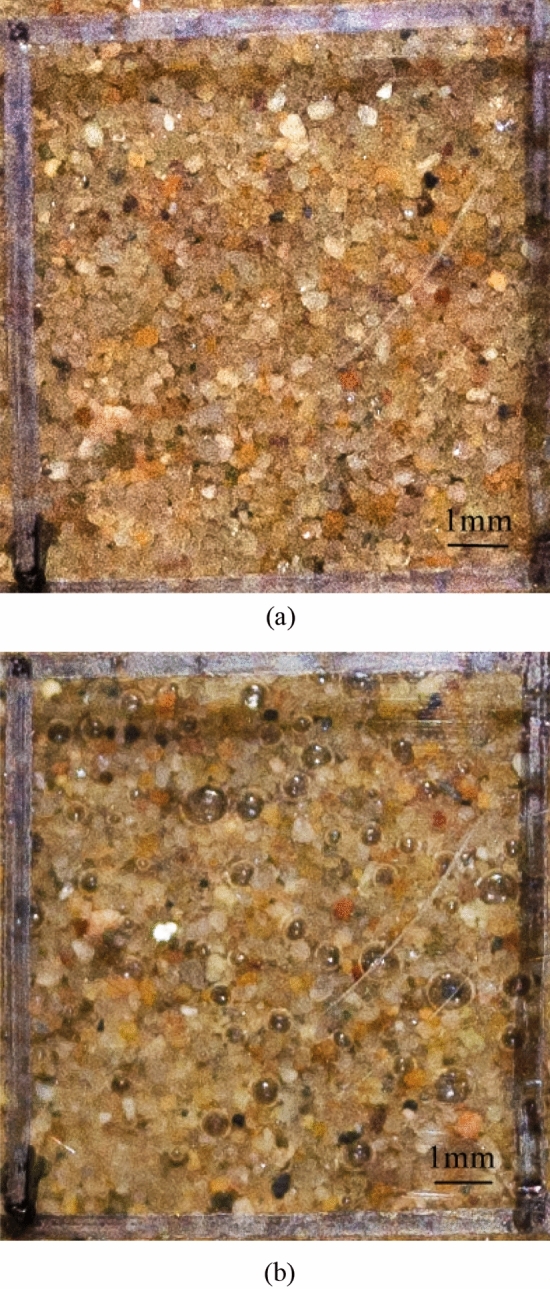
The change of bubbles.

In the second process, part of the biogas bubbles aggregated as shown in Fig. 12. Under the upward flow, the scale and quantity of bubbles become bigger and more bubbles at the top of the sample due to the seepage condition. The size of gas bubbles was approximately 0.2–0.5 mm, which was enough to occupy the pores of the sand and thereby reduce the permeability. Apart from the aggregation of the bubbles, bacteria, as organic matter particles, moved with seepage and reduced soil permeability. Bacteria may easily attach to the bubbles and form a rigorous blockage to consequently inhibit the subsequent migration of seepage. Bacteria plugging soil channels are highly common in oil exploration^36–38^.

## Conclusions

The long-term monitoring of sand specimens indicated that under hydrostatic conditions, the initial degree of saturation of 84.5% only slightly increased to 85.1%, after 72 weeks of monitoring. Small well-distributed biogas bubbles remained trapped for a long time.

The stability of soil under upward flow is the same as that under downward flow. When the hydraulic gradient was constant, the degree of saturation increased in a certain period of time and finally remained constant. Under downward flow conditions with a hydraulic gradient of 0.1, a change in the degree of saturation from 84.7 to 85.5% was observed. When the hydraulic gradient was equal to 0.2, the degree of saturation increased the most, and the saturation increment was 11.5% under upward flow conditions.

The coefficient of permeability decreased with time and finally remained constant when the hydraulic was constant. Under flow conditions with a hydraulic gradient of 0.2, a decrease in the coefficient of permeability from 0.00132 to 0.000176 cm/s was observed at 108 h. Biogas bubbles and bacterial plugging were the main reasons for the decrease in the coefficient of permeability and stability with the degree of saturation. By contrast, the dissolved phase was the main reason for the increase of the degree of saturation and the coefficient of permeability.

After 41,200 cycles of vibration, *V*_a_/*V*_ao_ decreased from 1.0 to 0.95, and a small number of bubbles spilled over the sand specimen. However, the degree of saturation of the sample decreased from 84.3% to 83.8% because of the settlement of the sample and the increase of its relative density during the test.
